# Comment on Hladky-Krage, B.; Hoffman, C.L. Expectations versus Reality of Designer Dog Ownership in the United States. *Animals* 2022, *12*, 3247

**DOI:** 10.3390/ani15101412

**Published:** 2025-05-14

**Authors:** Amanda J. Venturino, Katie M. Weaver, Cornelia Kraus, Jessica P. Hekman

**Affiliations:** 1 The Functional Dog Collaborative, Raymond, NH 03077, USA; 2 Environment, Health and Safety, University of Wisconsin-Madison, Madison, WI 53706, USA

## 1. Introduction

Designer dogs—the result of intentionally crossbreeding purebred dogs, and specifically poodle mixes (a cross between a poodle and a second breed)—have rapidly grown in popularity in recent years [1]. Research on the sociological, animal welfare, and veterinary implications of this phenomenon is still rare. In particular, ethical breeding is a topic of current relevance, and understanding what types of companions do well in households is a valuable field of study. Therefore, we were pleased to see the recent article published in Animals titled “Expectations versus Reality of Designer Dog Ownership in the United States” [2].

The authors investigated the motivation to own designer dogs and used owner questionnaires to evaluate the extent to which these dogs met owner expectations in various domains compared to purebred and mixed-breed dogs. The study focuses on doodles, the most popular subgroup of designer dogs. The authors hypothesize that owners select doodles due to expectations that do not align with reality. They list some possible expectations: (1) superior health in crossbred dogs, (2) better temperaments than parent breeds, specifically family-friendly temperaments, and (3) decreased shedding and allergenicity. (Note that item (3) does not apply to all designer dog types, and was also not specifically addressed in the study as a hypothesis, but is mentioned here as it was discussed in the Introduction.) Additionally, they suggest that designer dogs may be, more often than other types of dogs, (4) chosen based primarily on appearance. In their conclusion, they hypothesize that there is a disconnect between doodle owner expectations versus reality.

In this commentary, we suggest the following be considered with respect to the hypothesis and conclusions presented in Hladky-Krage and Hoffman’s article [2]. While we do believe this study contains relevant information regarding a subset of designer dogs, we here argue that additional results and analyses are needed to address the hypothesis. We also offer conclusions which we feel are more in line with the results presented, and make suggestions for further research on this emerging topic.

## 2. Better Health in Crossbred Dogs?

While the authors asked owners whether veterinary costs for their dogs met expectations (survey section I, question 1), the results based on this question were not reported in the paper, despite directly addressing the major hypothesis. Even a null hypothesis finding would constitute important evidence bearing on the authors’ hypothesis. The authors cite a study by Bellumori et al. [3] on inherited diseases as a reference that there was no difference between purebred and mixed-breed dogs in terms of the prevalence of 13 diseases. However, this study also showed that the prevalence was higher in purebred dogs for 10 diseases, but was only higher in mixed-breed dogs for one disease. Therefore, while indeed this paper does not show that crossbred dogs are inherently healthy simply due to being crossbred, it does actually provide evidence suggesting that there is a *decreased* risk of inherited disease in crossbred dogs compared to some breeds, which appears to be contrary to the authors’ intent in using it. There is a well-documented lifespan advantage of mixed-breed dogs compared to purebred dogs, especially when controlling for dog size, e.g., [4,5,6]. Additionally, Donner et al. [7] showed that purebred dogs were more likely to be at risk (i.e., homozygous) of inheriting common, testable genetic diseases than mixed-breed dogs, while mixed-breed dogs were more likely to be carriers (i.e., heterozygous). Other recent findings demonstrate that increased inbreeding is associated with increased overall morbidity [8] and decreased lifespan [9] across breeds, suggesting health benefits for outbred dogs. Therefore, we suggest that the authors’ statement, “the idea that crossbred dogs are healthier and impervious to genetic disease is not based on fact, as they can inherit the same conditions that purebred dogs can”, omits the evidence that crossbred dogs are, overall, at lower risk of genetic disorders than purebred dogs and have, on average, longer lifespans. This is the evidence that the public perception of benefits to crossbreeding is based on.

## 3. A Family-Friendly Temperament?

The survey distributed in this study did not explicitly test for a stable and family-friendly temperament in designer dogs. Hladky-Krage and Hoffman’s [2] results indicated that actual owner satisfaction was significantly higher with doodles than with the other groups tested, including owners with children in the home, which were twice as frequent as both purebred and mixed-breed dogs. This is consistent with the fact that 41% of doodle owners mentioned in the free-response section that their dog’s temperament did meet their expectations and 31% that they were good companion dogs. Moreover, the authors suggested that “most dog owners experienced few behavior problems” and that “there was no significant difference in behavior across the three dog types”. None of these reported results indicate unmet expectations of doodle owners regarding their dog’s temperament. To address the question of family-friendly temperaments more directly, a post hoc analysis testing if the effect of “children in the home” on behavior and satisfaction scores differs significantly among the three groups would be of interest.

Presentation of additional data may offer interesting insights into doodles’ suitability as pets. Specifically, findings from two relevant questions from Section I of the survey about expectations being met regarding exercise levels and overall behavior should be included, since they directly test the authors’ hypothesis. Because not all behavior problems are equally problematic in the family context, it would also be helpful to see group-specific ratings for the different behavior items of Section III. More specifically, nuisance barking was mentioned more often by doodle owners (25.5%) than non-doodle owners [purebreds: 18.5%; mixed breeds: 16.9%] in the qualitative free response section; a quantitative analysis of survey item 15 (“inappropriate barking”) from Section III of the questionnaire may be informative.

The final free response question, which invited participants to list up to three ways in which their dog met and did not meet their expectations, was optional. The paper only included discussion of a qualitative analysis for the responses provided by doodle owners. We suggest adding a comparison to the responses from purebred and mixed-breed owners, including numbers of answers from each group. We also encourage a note of caution when interpreting responses to this free-form and optional question.

## 4. Doodle Coat Maintenance and Allergenicity

Although assumptions about allergenicity were discussed in the Introduction, no questions regarding shedding or allergenicity were posed in the survey; therefore, the study did not directly address this question. About one-quarter of the doodle owners underestimated their dog’s maintenance level; the respective survey question is, likely inadvertently, missing in the Supplementary Materials, and these data would be useful to see. The results do show the importance of breeders explaining to new owners the significant grooming requirements associated with a longer, low-shedding coat.

## 5. Pre-Purchase Motivations of Doodle Owners

The authors conclude that doodle owners “may have been prioritizing aesthetics over health and behavior”. However, the results cited seem to support a more nuanced story. More doodle owners included the reasons “good with kids” (53.7%), “good companion breed” (83.2%), “generally healthy breed” (51.7%), and “breed suited to lifestyle” (69.8%) as more influential selection criteria than appearance (49.9%). Therefore, we suggest that appearance, while more frequently selected by doodle owners, was only one part of their decision, and in fact less influential than reasons relating to health and behavior.

## 6. Non-Doodle Designer Dogs

It is not clear if other types of designer dogs were omitted from the analyses or included in the “mixed-breed” category. In the Methods section, the authors state that “participants had the option of selecting ‘purebred dog’, ‘hybrid dog’, or ‘mixed breed not typically considered a hybrid’”, suggesting that the mixed-breed group in their analyses did not contain non-doodle designer dogs. However, in the results section, all dogs for which a survey was completed were assigned to one of three groups: 2191 surveys completed = 689 doodles + 854 other mixed breeds + 648 owners of purebred dogs. Non-doodle designer dogs were likely assigned to the mixed-breed category. We suggest that conclusions made from this survey be clearly stated as applicable to doodles only, rather than the designer dog phenomenon in general.

## 7. Literature and Language Use

In several instances, the authors cite non-scientific sources such as Smalley 2009 [10], an editorial from *Newsweek* magazine that is used to suggest that one-third of labradoodle puppies in each litter will have a high-shedding coat. However, genetic testing for the relevant coat type traits allows a more accurate prediction of, and selection for, coat types in multigenerational doodles [11,12], and is commonly used by doodle breeders. This source is also used to suggest that designer dogs make up an estimated 20% of dogs bred in puppy mills; we suggest that a more reliable source would be preferable for a peer-reviewed article. A considerable part of the introduction is based on small-scale qualitative interview studies with a very low level of evidence [13,14]. Language use throughout the introduction is pejorative, building a picture of a “recent fad” (i.e., by definition a short-lived phenomenon), regretted by the breeder who popularized labradoodles, emphasizing the numbers of designer dogs bred in puppy mills, and referring to a “misconception” (in both the Introduction and aim of the study) without showing that it actually is one. An alternative story could have been told which would describe the thirty-five year history of doodle popularity and longer history of other designer dog mixes, noting that, according to the cited source, four times as many purebreds are bred in puppy mills as designer mixes [10]. We suggest that responsible breeding is an important goal no matter what the mix or breed, and that popularity of particular types only drives the relative percentages of breeds or mixes in puppy mills, not the total number of dogs produced there.

## 8. Conclusions

The authors make strong statements in their discussion, such as, “Findings from this study indicate that owners of doodle dogs have certain expectations…not always rooted in reality…Doodle owners were also disproportionately influenced by the preconceived notion that doodles are good with kids and that they are generally a healthy breed, which is not consistently the case”, and “our results indicate that people acquiring doodles may make their acquisition decisions based on both aesthetics and misconceptions about the dogs, thinking them to be healthier and better with kids than other dog breeds”.

We offer a different set of conclusions, which we feel are better supported by the reported results: (1) owner satisfaction with doodles is higher than with other groups tested; (2) no indication was found that doodle owners experience more behavior problems with their dogs than owners of purebred or mixed-breed dogs; (3) the main owner dissatisfaction with doodles is in terms of grooming—owners did not understand the implications of a longer, low-shedding coat; (4); therefore, doodles do appear to make good pets, but it would be beneficial for the doodle-breeding community to find new and better ways to convey the significant grooming requirements associated with a longer, low-shedding coat.

We suggest these conclusions would be strengthened by the reporting and discussion of the missing data. We agree with the authors that this topic is important to prospective dog owners, and therefore wish to suggest potential future research questions. Direct comparisons of health and behavior of various designer dog breeds with their parent breeds would enable an analysis which, to some extent, controls for the dogs’ genetic background. Because some designer dogs (e.g., multigenerational goldendoodles) are far removed from the early-generation crosses, comparisons of such multigenerational dogs with their respective first-generation crosses would be of interest. With allergies being an important consideration in many families, research on the allergenicity level of dogs with different coat types is also needed.

We appreciate the significance and contribution this research provides to the designer breed community; there is a dearth of reporting on this particular topic. We invite Hladky-Krage and Hoffman to consider the above discussion and interpretation of their results with respect to their publication and welcome their feedback.

## References

[1] Benson J., Neff S., Tincher E.M., Nationwide Mutual Insurance Company Oodles of Doodles: Popularity and Health 2022. https://assets.ctfassets.net/440y9b545yd9/2xKSYWnUkxhb0tJGDLNdoI/fe9b401da2d73c6fef7c2a4bd4b8a49e/Nationwide_Oodles_of_Doodles_and_Cancer_Cross_Breed_White_Paper_2022.pdf.

[2] Hladky-Krage B., Hoffman C.L. (2022). Expectations versus Reality of Designer Dog Ownership in the United States. Animals.

[3] Bellumori T.P., Famula T.R., Bannasch D.L., Belanger J.M., Oberbauer A.M. (2013). Prevalence of inherited disorders among mixed-breed and purebred dogs: 27,254 cases (1995–2010). J. Am. Vet. Med. Assoc..

[4] Michell A.R. (1999). Longevity of British breeds of dog and its relationships with sex, size, cardiovascular variables and disease. Vet. Rec..

[5] O’Neill D.G., Church D.B., McGreevy P.D., Thomson P.C., Brodbelt D.C. (2013). Longevity and mortality of owned dogs in England. Vet. J..

[6] Yordy J., Kraus C., Hayward J.J., White M.E., Shannon L.M., Creevy K.E., Promislow D.E., Boyko A.R. (2020). Body size, inbreeding, and lifespan in domestic dogs. Conserv. Genet..

[7] Donner J., Anderson H., Davison S., Hughes A.M., Bouirmane J., Lindqvist J., Lytle K.M., Ganesan B., Ottka C., Ruotanen P. (2018). Frequency and distribution of 152 genetic disease variants in over 100,000 mixed breed and purebred dogs. PLOS Genet..

[8] Bannasch D., Famula T., Donner J., Anderson H., Honkanen L., Batcher K., Safra N., Thomasy S., Rebhun R. (2021). The effect of inbreeding, body size and morphology on health in dog breeds. Canine Med. Genet..

[9] Kraus C., Snyder-Mackler N., Promislow D.E. (2023). How size and genetic diversity shape lifespan across breeds of purebred dogs. GeroScience.

[10] Smalley S. (2009). Newsweek. A Designer Dog’s Life. https://link.gale.com/apps/doc/A197314055/AONE?u=nysl_we_caniscl&sid=AONE&xid=b1885666.

[11] Cadieu E., Neff M.W., Quignon P., Walsh K., Chase K., Parker H.G., VonHoldt B.M., Rhue A., Boyko A., Byers A. (2009). Coat Variation in the Domestic Dog is Governed by Variants in Three Genes. Science.

[12] Hayward J.J., Castelhano M.G., Oliveira K.C., Corey E., Balkman C., Baxter T.L., Casal M.L., Center S.A., Fang M., Garrison S.J. (2016). Complex disease and phenotype mapping in the domestic dog. Nat. Commun..

[13] Beverland M.B., Farrelly F., Lim E.A.C. (2008). Exploring the dark side of pet ownership: Status-and control-based pet consumption. J. Bus. Res..

[14] Power E.R. (2012). Domestication and the dog: Embodying home. Area.

